# Planning care for nursing-home residents: cognitive impairment, functional independence, and accommodation services in nursing homes in the city of Zagreb

**DOI:** 10.3325/cmj.2025.66.265

**Published:** 2025-08

**Authors:** Nada Tomasović, Mrčela, Zrinka Mach, Marija Kušan Jukić, Marija Penava Šimac, Branko Kolarić

**Affiliations:** 1 Department of Public Health Gerontology, Andrija Štampar Teaching Institute of Public Health, Zagreb, Croatia; 2 University of Split, Faculty of Health Sciences, Split, Croatia; 3 Alma Mater Europaea University, Maribor, Slovenia; 4 Croatian Society for Chronic Diseases, Zagreb, Croatia; 5 Department for Mental Health and Addiction Prevention, Andrija Štampar Teaching Institute of Public Health, Zagreb, Croatia; 6 Ministry of Labor, Pension System, Family and Social Policy, Zagreb, Croatia; 7 School of Medicine, University of Rijeka, Rijeka, Croatia; 8 Academy of Medical Sciences of Croatia, Zagreb, Croatia; * The first two authors contributed equally.

## Abstract

**Aim:**

To assess whether the Mini-Mental State Examination, second edition (MMSE-2), scores were associated with the category of functional independence of nursing-home residents and the level of accommodation services they received.

**Methods:**

This cross-sectional study enrolled 248 participants older than 65 residing in five county-owned nursing homes in the city of Zagreb from 2017 to 2019. Cognitive status was assessed with the standard version of the MMSE-2, and the level of functional independence with the modified Barthel scale index. We collected data on demographic, clinical, and social characteristics, including the level of accommodation services (I-III).

**Results:**

MMSE scores were associated with the category of functional independence (χ^2^ = 21.11; *P* < 0.001) and the level of accommodation services provided to nursing-home residents (χ^2^ = 38.87; *P* < 0.001).

**Conclusion:**

Cognitive impairment should be determined in nursing-home residents to facilitate the planning of long-term institutional care and the implementation of the prescribed standards based on the level of accommodation services.

The organization of long-term care for the elderly, including planning and cost rationalization, is usually determined by state policies (1). The planners of such care face challenges in terms of demographic indicators of an aging society, a growing share of people in deep old age, and increased life expectancy (2-5). In addition, people in their eighties and nineties who need long-term integrated health and social care often live alone and are functionally disabled (2,6-8). The organizational concepts and intersectoral gerontological approach are especially important in ensuring and improving the quality of long-term care for the elderly (9). Furthermore, to ensure that long-term institutional care meets legislation and regulations, there should be an in-depth analysis of gerontological and public-health indicators of health care needs and functional ability of residents (10,11).

In a German study, factors associated with placement in a nursing home in deep old age were marital status, social isolation, depressive symptoms, increased cognitive impairment, and a higher level of frailty (12). According to Luppa et al (13), the strongest predictor of institutionalization was dementia, followed by under-assessed health status and polypharmacy. Multimorbidity, including cardiovascular diseases, dementia, and anemia, represents an increased risk for placement in a nursing home (14). Factors that predict placement in a nursing home for patients with dementia are health status, sociodemographic characteristics, and caregiver-related factors (15). In people suffering from dementia, cognitive disorders interfere with everyday activities and compromise their quality of life (16). Finally, an important predictor of placement in a nursing home is the individuals’ ability to perform basic daily life activities (6,17,18).

On average, about 5% of the elderly receive long-term care in institutions/facilities in the EU (19). In 2022, Croatian nursing homes accommodated 17 269 persons aged 65 and over, which amounts to 2% of the total elderly population, distributed in state-owned nursing homes (n = 3), county-owned nursing homes (n = 45), and other homes for the elderly and severely ill adults (n = 120) (20). The main reasons for placement in Croatian nursing homes are illness and infirmity, disability, loneliness, inadequate living conditions, and dysfunctional family relationships (21).

Pavlovic et al investigated the correlation between nursing-home residents’ cognitive status and their functional abilities (22). Our study assessed whether cognitive status was associated with the category of functional independence of nursing-home residents and the level of accommodation services they received in order to inform planning the organization of long-term institutional care in Croatia.

## PARTICIPANTS AND METHODS

### Setting and study participants

This study is a continuation of our research program looking into the health care of nursing-home residents in Zagreb, Croatia (23-26). This cross-sectional study enrolled 150 residents of three county-owned nursing homes (Maksimir, Pešćenica, Sv. Josip) in the period 2017-2018 (23) and 98 residents from the nursing home Sv. Josip and two additional homes (Dubrava and Ksaver) in the period 2018-2019 (24). The overall number of participants was 248.

The total number of residents in five county-owned nursing homes according to the level of accommodation services was as follows: level I – 567, level II – 280, and level III – 545. Level-IV services were provided for 105 residents, who were not included in this study. The participants were consecutively enrolled. Every other resident in alphabetical order for each level of accommodation services (I to III) was offered to participate in the study (25). The participants were then randomly enrolled in the study as follows: 100 participants for level I, 66 for level II, and 82 for level III. The required sample size was established based on pilot research.

The study was carried out in accordance with the Helsinki Declaration and was approved by the Ethics Committee of the Andrija Štampar Teaching Institute of Public Health. The consent for conducting research in nursing homes was obtained from the City of Zagreb's Office for Social Protection and Persons with Disabilities of Croatia. All participants were informed about the study protocol and signed an informed consent for participation.

The study was performed in two parts. In the first part (2017-2018), we enrolled mostly functionally independent residents of nursing homes (level-I accommodation services) and in the second part (2018-2019) we enrolled the residents who depended on the help of others (level-II and -III accommodation services). There was no overlap between the participants from the two parts of the study.

### The level of accommodation services in nursing homes

The accommodation for the elderly is provided in a defined intensity according to Article 68, item 2 of the Regulations on Criteria for the Provision of Social Services based on health impairment, functional ability, and required care for basic life needs (11). Level-I services are provided to functionally independent users, level-II services to partially dependent users who need assistance in meeting their basic needs, and level-III services to residents who need assistance in meeting all their needs in full. Level-IV services are provided to residents who are completely functionally dependent and require help and supervision of another person in meeting all their needs in full due to Alzheimer’s dementia or other dementias (medium/moderately severe) (11).

### The inclusion and exclusion criteria

The inclusion criteria were residence in one of the five selected county-owned nursing homes in Zagreb (Sv. Josip, Maksimir, Pešćenica, Dubrava, and Ksaver), age 65 and over, and agreement to participate in the study. The exclusion criteria were serious health problems that could affect the participant’s ability to fill out the questionnaire and provide the informed consent, such as severe mental disorders and severe cognitive impairment (dementia), but also high fever or disturbance of consciousness.

### Measuring instruments

This study was a part of the Andrija Štampar Public Health Institute program entitled “Improving the Quality of Life and Health Protection of Nursing Home Residents by Designing a Guide for Active Healthy Ageing” in Zagreb (2017-2019), which employed the modified Barthel scale index and Mini-Mental State Examination, second edition (MMSE-2), and collected data on demographic and clinical/social characteristics of the participants (23-29).

The standard version of the MMSE-2 was used, with scores of up to 24 points suggesting dementia; 25-27 suggesting mild cognitive impairment (MCI), and 28-30 indicating no cognitive impairment (27-31). The MMSE is one of the most commonly used scales for assessing cognitive deficits, consisting of 10 tasks and a maximum score of 30 (28,29). The tasks are mainly aimed at testing memory, speech, and attention, while other cognitive domains are less well represented or not represented at all. The threshold values of the MMSE test for suspecting cognitive impairment can be affected by age and education (28,29).

The participants were classified into five categories of functional independence based on the Barthel scale index modified according to Shah, Vanclay and Cooper (MBI) (32), as follows: 1) 100 – complete independence, 2) 91 to 99 – slight dependence, 3) 61 to 90 – moderate dependence, 4) 21 to 60 – severe dependence, and 5) 0 to 20 – total dependence on other people's help. The index measures the degree of functional independence in personal care and mobility in ten common daily activities (25,32). The survey was carried out by experts specially trained to work with elderly people (a physician, gerontologist, and nurse, who are not the employees of any nursing homes).

### Statistical analysis

The results are presented as frequencies or medians with interquartile ranges. The differences between the groups were assessed using a χ^2^ test or Kruskal-Wallis test. The correlations were evaluated using the Spearman correlation coefficient. Rho values ranging from 0 to 0.25 or from 0 to −0.25 point to an absence of correlation; values from 0.25 to 0.50 or −0.25 to −0.50 indicate poor correlation; those from 0.50 to 0.75 or −0.50 to −0.75 indicate moderate to good correlation; and those from 0.75 to 1 or from −0.75 to −1 indicate good to excellent correlation (33). *P* values <0.05 were considered significant. The analysis was performed using Statistica 12 (TIBCO software Inc, Palo Alto, CA, USA).

## RESULTS

There were three times as many female as male participants. The greatest number of participants was in the 85+ age group (χ^2^ = 67.00; *P* < 0.001) (Table 1). Most participants had an MMSE-2 score of <24; there were significantly more participants with an MMSE-2 score <24 than those with an MMSE-2 score of 28-30 (χ^2^ = 9.85; *P* = 0.007). Overall, 247/248 participants completed the MMSE-2 test. Most participants were completely independent; there were significantly more completely independent participants than those belonging to other categories (χ^2^ = 104.58; *P* < 0.001). The most common level of accommodation service was level I. The largest number of participants completed secondary education (χ^2^ = 84.00; *P* < 0.001) (Table 1).

**Table 1:** Demographic and clinical/social characteristics of study participants

Variable	Category	n	%	χ^2^	p
Sex	female	186	75.00	62.00	<0.001
	male	62	25.00		
Age group	65-74 (early old age)	22	8.87	67.00	<0.001
	75-84	110	44.35		
	85+	116	46.77		
MMSE-2* test scores	<24	101	40.89	9.85	0.007
	25-27	85	34.41		
	28-30	61	24.70		
Functional independence (modified Barthel scale index)	5 – complete independence	98	39.52	104.58	<0.001
	4 – slight dependence	45	18.15		
	3 – moderate dependence	69	27.82		
	2 – severe dependence	3	1.21		
	1 – total dependence	33	13.31		
Level of accommodation services	I	100	40.32	7.00	0.030
	II	66	26.61		
	III	82	33.06		
Level of education	primary	80	32.26	84.00	*P* < 0.001
	secondary	112	45.16		
	bachelor	22	8.87		
	master’s or higher	34	13.71		

*Mini-Mental State Examination, second edition.

Complete independence was most frequently observed among respondents with an MMSE score of 28-30 (n = 36; 59.02%) and least frequently among respondents with an MMSE score <24 (Table 2). There were significant differences in the participants' category of functional independence in relation to the MMSE score (χ^2^ = 21.11; *P* < 0.001). The median functional independence score for participants with an MMSE score <24 was 3.00 (IQR = 3.00-4.00), for participants with an MMSE score of 25-27, it was 5.00 (IQR = 4.00-5.00), and for participants with a score of 28-30 it was also 5.00 (IQR = 3.00-5.00). *Post-hoc* analysis revealed significant differences in functional independence across all MMSE score categories (*P* < 0.001) (Table 2).

**Table 2:** Categories of functional independence (modifed Barthel scale index [MBI]) and Mini-Mental State Examination (MMSE-2) test scores in nursing-home residents

	Functional independence (MBI)												
MMSE-2 test scores	5 – complete independence		4 – slight dependence		3 – moderate dependence		2 – severe dependence		1 – total dependence		Median (IQR)	χ^2^*	P
	n	%	n	%	n	%	n	%	n	%		21.11	<0.001
<24	24	23.76	18	17.82	35	34.65	1	0.99	23	22.77	3.00 (3.00-4.00)
25-27	38	44.71	20	23.53	19	22.35	2	2.35	6	7.06	4.00 (3.00-5.00)
28-30	36	59.02	7	11.48	15	24.59	0	0	3	4.92	5.00 (3.00-5.00)

*Kruskal-Wallis test.

The most prevalent level of accommodation services among participants with an MMSE score below 24 was III (Table 3). This category was 1.87 times more common than among participants with an MMSE score of 25-27, and 4.40 times more common than among participants with a score of 28-30. MMSE scores were associated with the level of accommodation services (χ^2^ = 38.87; *P* < 0.001) (Table 3).

**Table 3:** The level of accommodation services and Mini-Mental State Examination (MMSE-2) test scores in nursing-home residents

	Level of accommodation services							
MMSE-2 test scores	I		II		III		χ^2^	p
	n	%	n	%	n	%		
<24	20	19.80	30	29.70	51	50.50		
25-27	43	50.59	19	22.35	23	27.06		
28-30	37	60.66	17	27.87	7	11.48	38.87	<0.001

There was a significant weak positive correlation between MMSE-2 score and the level of functionality on the MBI scale (rho = 0.35; *P* < 0.001). There was also a significant weak negative correlation between MMSE-2 score and the level of accommodation services (rho = -0.44; *P* < 0.001).

## DISCUSSION

In this study, participants with lower functional independence had MMSE test scores indicating suspect dementia (<24). Participants with MCI were most often fully functionally independent. The MMSE-2 score was also associated with the level of accommodation services. Participants with an MMSE-2 score of 25-27 most often received level-I social services, while those with an MMSE-2 score <24 received a higher level of accommodation services. These results indicate that the planning of accommodation services in nursing homes must take into account the prevalence of residents with suspected cognitive impairment suggesting dementia (MMSE-2 score <24).

Our results indicate the importance of screening residents for cognitive impairment and functional ability, which, in addition to their health needs, are key gerontological indicators in nursing homes. Consequently, the family medicine specialist team needs guidelines for a rational diagnostic work-up for cognitive impairment in the elderly in institutional and non-institutional care. Screening for cognitive impairment in the elderly should consider the so-called risk profile of the patient combined with an individual gerontological approach and acceptance of screening by the patient (16,34). However, such screening is controversial as it may lead to the occurrence of depressed mood, suicidal thoughts, and discrimination or social stigma (16,34).

Not all people with MCI will develop dementia, but timely recognition of MCI contributes to the quality of patient care. It is important to distinguish between physiological changes associated with aging such as mild forgetfulness (misplacing things, difficulty recalling words) and dementia (16). It is also important to maintain and possibly improve the level of functional ability because it can mediate the effects of age on global cognitive function in the elderly (35).

de Oliveira et al (36) found an interrelation between the patient's cognition, functionality, and behavior in all stages of Alzheimer’s disease. However, a study among patients aged 65 years and older in a subacute care unit in Australia did not find a correlation between patients' cognitive status, as assessed by MMSE score and the Montreal Cognitive Assessment, and functional performance measured by the Functional Independence Measure questionnaire (37). Pavlovic et al confirmed a significant weak correlation between nursing-home residents’ cognitive status and functional abilities, with 60% of respondents having cognitive disorders (22).

In our study, about 41% of participants scored <24 points on the MMSE-2 test, which points to the need for organizational prioritization in long-term care. Contrary to this, gerontological and public health analysis in ten county-owned nursing homes in Zagreb showed that only 2.29% of residents used level-IV accommodation services in 2019, 1.07% in 2020, and 0.99% in 2021 (38). This finding indicates that the estimated levels of accommodation services need to be more frequently revised, especially for individuals with a verified diagnosis of Alzheimer’s disease and other dementias.

According to the Regulations on Criteria for the Provision of Social Services (11), the prescribed standards for space, equipment, and professional workers must be met for each level of accommodation services. Consequently, specialized departments for elderly patients suffering from Alzheimer’s disease and other dementias are being organized in certain nursing homes in the City of Zagreb (39). The Regulations emphasize additional and specific conditions for the provision of level-IV accommodation services to functionally dependent users with Alzheimer’s disease or other causes of dementia (medium/moderately severe stage of the disease) (11).

There is a noticeable difference in personnel standards according to the levels of accommodation services for the elderly, which ensures the quality of the health and social services provided in nursing homes (11). The adherence to the prescribed personnel norms in the health care of the elderly supports the quality of care, preserves workers’ health, and establishes a culture of safety (9). Consequently, there are numerous reasons for determining and monitoring cognitive impairment in nursing-home residents. This intention also complies with the legal standards regarding space and equipment necessary for the provision of accommodation services to elderly and seriously ill adults, along with the minimum number of workers per number of service units/number of residents.

The limitations of our study include the geographically limited sample and the exclusive use of the MMSE-2 test for screening cognitive impairment. However, the MMSE is the most commonly used and internationally accepted scale for the assessment of cognitive deficits (16,28). Furthermore, although the study was completed in 2019, the legislation has not changed in the meantime, and no new protocols have been introduced related to the monitoring of the cognitive status of nursing-home residents in Croatia.

In conclusion, this study showed that the cognitive status of nursing-home residents was associated with the level of accommodation services (from I to III). Our results point to the necessity of designing guidelines for the identification and monitoring of cognitive impairment of the elderly in nursing homes. Further gerontological and public health analyses are needed regarding the norms and standards in the health care of the elderly.
